# A device for volatile organic compound (VOC) analysis from skin using heated dynamic headspace sampling

**DOI:** 10.1088/1752-7163/adccef

**Published:** 2025-05-06

**Authors:** Flore M Hervé, Eva Borras, Patrick Gibson, Mitchell M McCartney, Nicholas J Kenyon, Cristina E Davis

**Affiliations:** 1 Mechanical and Aerospace Engineering, UC Davis, Davis, CA, United States of America; 2 UC Davis Lung Center, Davis, CA, United States of America; 3 VA Northern California Health Care System, Mather, CA, United States of America; 4 Department of Internal Medicine, UC Davis, Sacramento, CA, United States of America

**Keywords:** skin, VOCs, sampling, mass spectrometry, gas chromatography

## Abstract

Human skin is an important source of volatile organic compounds (VOCs) offering noninvasive methods to gain clinical metabolite information. This work was focused on the development of a skin sampling device based on a dynamic headspace sampling method with the addition of temperature to increase VOC metabolite recovery. The device preconcentrates skin VOC emissions onto a sorbent substrate, which can either be preserved for offline analysis or attached to a real time sensor downstream. In this work, skin VOC samples were analyzed offline using thermal desorption-gas chromatography-mass spectrometry. A list of 10 common skin VOCs was pre-selected to optimize parameters of sampling time, sampling temperature, and sorbent selection. Overall, this study highlights an effective skin VOC sampling technology with a heating dimension (40 °C, rather than 30 °C or no heating) with a sampling time of 15 min (rather than 5 or 30 mins) and onto Tenax TA sorbent (rather than PDMS), which collectively increases the recovery of compounds with lower vapor pressure and decreases the observed variability in skin VOC measurements. Finally, a list of 79 skin VOC compounds were detected and identified within a cohort of 20 young, healthy volunteers.

## 1. Introduction

As the body’s largest organ, skin represents more than 15% of our body weight [1], and is readily accessible as a major source of human volatile organic compound (VOC) emissions. The emitted VOCs are mainly derived from sweat, secreted from eccrine and apocrine glands, and sebum, which is secreted by sebaceous glands [2, 3].

These different glands, commensal bacterial and fungal flora that form the skin microbiome are unevenly distributed within the skin [4] leading to different VOC patterns based on the area of sampling as reported in studies for the axillary [5], forearm [6] and whole-body [7]. In addition, VOCs from an individual vary with diet [8], emotional state [9], hormone level [10, 11], age and other environmental factors.

Numerous studies have been reported on human scent [12, 13] and its relation to neurodegenerative diseases [14], wound infection [15], mosquito attraction [16], and pollutant exposure [17]. Because emissions reflect metabolic conditions of an individual, analysis of skin VOCs offers a non-invasive biochemical body probe for disease and disorder diagnosis.

A complex mixture of aldehydes, ketones, alkenes, amines, alcohols, and hydrocarbons has been detected from skin [18], but in low concentrations; therefore, an enrichment step before quantitative and/or qualitative analysis is commonly required. Advances in skin collection devices and sensitive analytical techniques have been achieved using direct sampling (contact) [19, 20] or indirect sampling, referred to as headspace (HS) sampling. The direct sampling approach uses a sorbent in contact with the skin. The advantage of this approach is that it provides good extraction capability for both volatile and non-volatile analytes. The main drawback is that oily residue from skin secretions or cosmetics (lotions) may adhere to the sampling device resulting in matrix contamination.

The indirect or HS sampling method prevents contamination from oils or fats present on the skin. Two different methods can be used for this: (a) static HS sampling [21] where the sample is allowed to emanate into an enclosed housing with a constant volume for a given time until the HS volume of the housing is saturated with the vapor analyte; or (b) dynamic HS sampling (DHS) involving a continuous flow of carrier gas passing through a specific volume of a stationary sample for a given time. DHS is widely used in VOC sampling of many matrix types, both biological and inorganic, is recognized to allow a higher compound recovery compared to static sampling [22]. Furthermore, it is recognized that increasing the sampling temperature encourages evaporation of VOCs from the matrix surface into the HS volume, improving recovery in terms of the number and abundance of measured chemicals.

The aim of the present study was to design a non-invasive DHS device with the addition of temperature for skin VOCs analysis. DHS is known to increase the detection of VOCs from an otherwise static environment, and is used to sample VOCs from biofluids like urine. No such alternative existed for sampling skin VOCs, a gap our technology fulfills. The device was designed for sampling of the posterior forearm, though its design allows for scaling to accommodate other skin areas of interest. The sampling time, temperature, and sorbent type were optimized to obtain the maximum VOC signal from the skin. We observed baseline skin VOC measurements from a cohort of 20 healthy volunteers with no reported health conditions. The method developed in this work provides a new, simple approach for the collection and analysis of skin VOCs for human health research.

## 2. Materials and methods

### 2.1. Subject enrollment and skin VOC sample collection

Healthy volunteers were recruited (*n* = 20, age range 20–45 years, with 13 males) having given their informed consent under an human subjects, IRB-approved study (UC Davis IRB #1859852).

To minimize contributions from exogenous sources, a skin pre-treatment step was included in the sampling protocol. Participants washed their forearm with ethyl alcohol and waited 30 min prior to beginning sampling. No special dietary regimes were applied.

Skin sampling was performed in the same location at UC Davis under a temperature-controlled environment (20 °C).

### 2.2. Description of the skin VOC sampling patch

**Figure 1.**
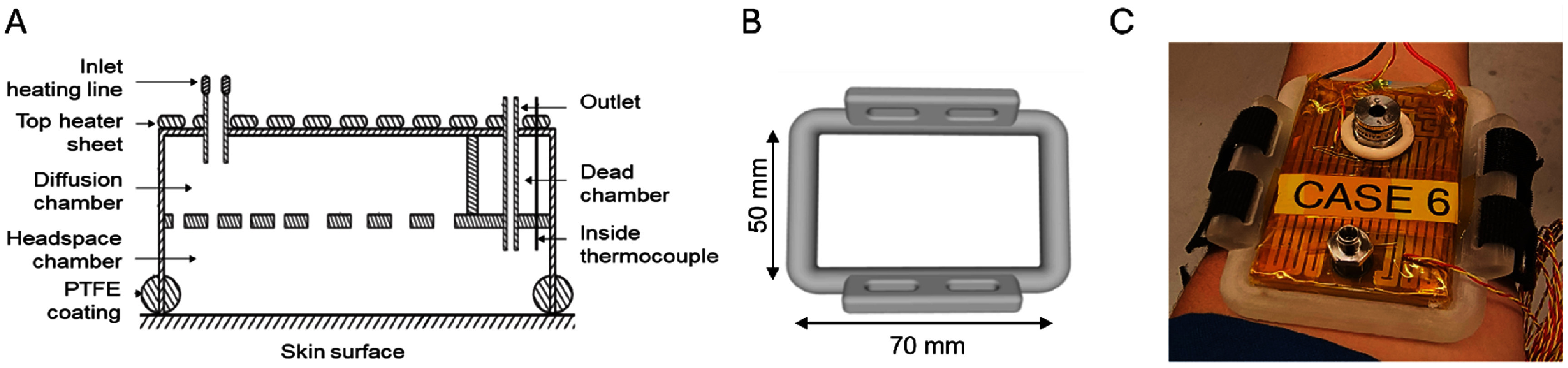
Developed device to collect skin VOC emissions. (A) Cross-sectional schematic of the sampling patch showing one inlet and one outlet, three chambers (diffusion, headspace, and dead), separation between each chamber, two heating systems (inlet line and top sheet), and a gasket made of biocompatible PTFE, which is the only portion to come in direct contact with the skin. (B) 3D model of the gasket to install around the patch for secure placement and sealing with the skin surface. (C) Picture of the sampling system (patch, Tegaderm adhesive, and the gasket held in place with Velcro straps) onto the posterior forearm.

Volatiles emitted by human skin were collected using our in-house custom designed wearable housing, henceforth to be referred to as the skin VOC sampling patch (figure 1). The forearm was selected as sampling area for volunteers’ comfort and convenience of sampler installation, and for the high emission rate of VOCs from forearm compared to other surfaces like the upper arm [23]. Briefly, the patch is secured onto the posterior forearm via a gasket and 3 M™ Tegaderm™ dressing (figure 1(C)). A one-way flow of filtered air (0.22 *µ*m filter) ensures a-free background atmosphere, while skin VOCs are isolated and collected into the housing, or HS. To increase VOC emissions from the skin, the temperature of the patch atmosphere can be increased above ambient up to 40 °C via a heating system, safe to prevent injuries to the skin. As a redundant safety measure, an electrical circuit interrupts power to both the patch inlet and surface heating elements if an independent thermocouple registers a temperature exceeding a safety threshold. Skin VOCs from the patch collect onto a chemical sorbent for preconcentration prior to chemical analysis. A detailed description follows.

The patch is fabricated from a 1 mm thick sheet of polyvinylidene fluoride (PVDF) (CS hyde company, 32–30 F-26 V) with housing outer dimensions of 4 cm width × 6 cm length × 1 cm height (figure 1(A)). Providing the advantages of DHS, the patch is composed of an inlet and outlet allowing continuous flow through a chamber design for HS formation. Two other chambers are formed, one to improve the distribution of the carrier gas called the diffusion chamber and a second dead chamber to reduce unnecessary volume in design. A complete design of the patch is shown (supplemental figure 1). To stabilize the patch onto the skin and distribute the clamping force, a 3D printed gasket made of latex-free resin (Formlab, RS-F2-PRGR-01) surrounds the edges of the bottom of the patch coated with PTFE and includes adjustable Velcro straps that secure the patch around the forearm (figure 1(B)). To prevent background VOC contamination, the patch is sealed to the forearm by a transparent waterproof adhesive (3 M™, Tegaderm™), commonly used in clinical settings. The inlet and outlet for the carrier gas are located on the top surface of the patch. The flow is generated by a disc pump (Ion Science, XP-S2-028) affording flow rates between 0 and 1000 ml min^−1^; the flow rate used in this study was 100 ml min^−1^, which was calibrated prior to each sample being taken using an external flow meter (Restek, 22 656).

### 2.3. Heating of the sampling patch HS

Increased HS temperatures can improve collection of compounds with low volatility. Thus, the system design includes heating of the inlet gas and of the housing itself. Initial testing indicated that direct flow of heated gas into the chamber had potential to cause high local temperatures on the forearm skin, leading to potential safety issues. To ensure an even and safe distribution of temperature on the skin surface, a second chamber fabricated with PDVF was added inside of the patch. It was perforated along its entire length with 4 lines of 2 mm diameter holes and 4 lines of 5 mm diameter holes. This divides the patch interior into a diffusion chamber with dimensions measuring 40 mm *L* × 38 mm *W* × 5 mm *H* and a HS chamber formation of 58 mm *L* × 38 mm *W* × 5 mm *H* (figure 1(A)).

Two heating systems were implemented, first with a heated stainless-steel line at the inlet allowing the heating of the carrier gas (filtered air) and a second custom designed heater (Fluxteq) was placed on the top of the patch to decrease the heat loss through the housing material. The heaters are powered by external power supply (Keithley, 2231 A-30-3) and controlled by external temperature controller (Omega, CN7500). A thermocouple was incorporated inside of the patch, next to the outlet to monitor the HS temperature in real time.

Experiments indicate that an internal HS temperature of 30 °C and 40 °C could be achieved by controlling the inlet line at 50 °C and 60 °C and the patch surface heater at 40 °C and 55 °C, respectively. The maximum temperature experienced by the skin is 40 °C, following the regulations for skin temperature sensitivity. Additionally, an electronic safety system was implemented into the sampling system, cutting off the power to the heaters if the inside patch temperature reached 42 °C and not restoring power until the temperature decreased to 38 °C.

### 2.4. Preconcentration of skin VOCs onto sorbent

Skin VOC emissions are typically in low concentrations, therefore preconcentration via a chemical sorbent is preferred prior to chemical analysis. As VOCs are emitted by the skin, the carrier gas directs them out of the patch via the outlet to a downstream location where any type of sorbent or VOC capture system can be attached.

In this work, two commercially available sorbents were tested: Twisters, or polydimethylsiloxane (PDMS)-coated stir bars (10 mm in length, 0.5 mm in film thickness; Gerstel GmbH), and thermal desorption tubes containing Tenax TA sorbent (Gerstel, 02081000500). PDMS was used for the optimization of sampling time and sampling temperature. Then, using the optimal sampling time and temperature, we compared PDMS to Tenax TA sorbent. PDMS Twisters were conditioned first in a solvent mix of acetonitrile: methanol (1:4) overnight, then dried for one hour under the hood and heated to 250 °C for 2 h under nitrogen before use. Tenax TA was conditioned under nitrogen at 250 °C for 2 h before use. To collect skin VOCs, Twisters were placed into a hollow glass tube connected to the sampling patch outlet. Similarly, the Tenax thermal desorption tubes were directly connected to the sampling patch outlet. After collection, sorbent substrates are removed, sealed, and preserved (refrigerated) until chemical analysis.

### 2.5. Gas chromatography-mass spectrometry (GC-MS) and data analysis

Once captured onto sorbent, skin VOC samples were desorbed within a thermal desorption unit (TDU2, Gerstel) and cryofocused using a Cooled Injection System (CIS4, Gerstel) in the injector of an Agilent series MSD 5975 gas chromatograph. Sample desorption was performed at 250 °C for 2 min in splitless mode. Separations were performed on an HP-5 ms column (30 m × 0.25 mm × 0.25 *μ*m do, Supelco, Bellefonte, PA, USA) with helium carrier gas at a constant flow rate of 1 ml min^−1^ . The initial GC oven temperature was set to 30 °C for 1 min, after which the oven was heated at a rate of 5 °C min^−1^–150 °C, followed by a rate of 2.5 °C min^−1^–200 °C with a hold for 2 min, and a final temperature ramp at a rate of 15 °C min^−1^–270 °C. The single quadrupole mass analyzer, Agilent series MSD 5975, was operated at a scan rate of 3.94 Hz and scanned a range of 35–400 m/z. An ionizing energy of 70 eV was used, and the ion source temperature was maintained at 230 °C.

Deconvolution of mass spectral data was performed using Agilent MassHunter Quantitative Unknowns Analysis software v.10.2 (Agilent Technologies). The identification of compounds was performed using the National Institute of Standards and Technology library (NIST 2023) to >80% match factor and was supported by retention index (RI) matching (tolerance of ⩽10 RI units), for which a standard mixture of saturated alkanes (C_7_–C_30_, Sigma Aldrich) was used. Based on the detected and identified compounds, a custom library was built using Agilent MassHunter Quantitative software v.10.2 (Agilent Technologies), which was then applied to all samples to obtain a peak table, denoting the abundance of each compound in all samples.

The peak table was exported and analyzed using Excel, MATLAB R2023 and PLS Toolbox V8.6.2. Any artefact coming from the background housing system and sorbent were subtracted from the peak list. Post processing, statistical analysis, and visualization were then performed. Data were pretreated by normalization (−1 norm, area = 1) transformation log 10, then Pareto scaling, and finally mean centering.

To assess the significance of sampling parameter efficiency, compounds intensity (integrated peak area) was compared using Wilcoxon’s rank. Any *p* value <0.05 were considered to have a significant difference. Partial least-squares discriminant analysis (PLS-DA) was later used as a supervised technique that uses compounds information to maximize the discrimination between groups of samples. PLS-DA models maximum covariance between datasets and defined sample class, separating the different groups studied based on their metabolite features. Models were evaluated using multiple test-validations by randomly dividing the samples into two cohorts composed of 66% training and 33% validation subjects. Potential biomarkers to differentiate case from groups were selected according to the variable importance in the projection (VIP) values. VIPs summarize the contribution that each feature makes to the model, and values higher than 1 are considered relevant biomarkers.

In this study, we compared sampling times and temperatures to optimize skin VOC capturing on PDMS. Then, we compared two sorbent types, a PDMS-coated stirbar (Twisters) and Tenax TA packed thermal desorption tubes. Control samples were also collected, including instrumental blanks and the background of an empty skin patch attached to a glass slide, to exclude exogenous compounds from analyses. All sampling in this study was performed in the same room location.

## 3. Results and discussion

In this work, we present a novel device to capture VOC emissions from skin. Although DHS for skin VOCs has been already described in literature, the skin VOC sampling housing presented (figure 1), to our knowledge, is the first to incorporate heat to encourage evaporation of skin VOCs, increasing analyte capture and recovery, as demonstrated herein. Results regarding the optimization of the sampling time, sampling temperature, and a comparison on how effectively the two sorbents capture skin VOCs are presented below. The skin VOC profile obtained from 20 volunteers with no known diseases or disorders is described.

### 3.1. Sampler design and comfort

The two heater systems offer control of interior HS chamber temperature within a range from ambient to 40 °C, and the inside thermocouple provides a real time temperature reading of the housing HS. Furthermore, the design of two layered chambers, diffusion and HS, ensures a homogenous distribution of heat onto the skin. User feedback raised no criticisms, confirming that the patch was light and that patch placement was comfortable for extended wear (in this study, no more than 1 h). No subjects reported experiencing high temperature sensation, burning, irritation, or extra dryness with the heated sampler, even at the highest temperature (40 °C) for more than 15 min. In fact, subjects reported they would not have known heating was occurring had they not been informed beforehand.

### 3.2. Panel of skin VOCs for method optimization

Before optimizing method parameters, we first created a panel of 10 skin VOCs. The selection of compounds was made in two steps, first based on the literature that reported VOCs found in skin [18, 24], then after preliminary skin analysis performed in our laboratory. We selected compounds found consistently in our healthy volunteers. Overall, the list contains a wide variety of compounds ranging between 6 and 19 carbons, including aldehydes, ketones, alkanes, esters, and aromatic hydrocarbons (table 1).

**Table 1.** Selected Compounds for sampling parameter optimization. Key information provided includes compound name, CAS number, formula, molecular weight, emission rate, boiling point, vapor pressure, Henry’s law constant and polar surface area. Emission rates from this table were extract from previous reports [21, 25].

Compound name	Cas number	Formula	Molecular weight (Da)	Chemical Family	Emission rate range [fmol × cm^−2^ × min − 1]	Boiling Point at 760 mm Hg (in °C)	Vapor pressure at 25 °C (in mmHg)	Henry’s Law Constant (in mol/(m^3^Pa))	Polar surface area (in Å^2^)
Heptane	142‐82‐5	C_7_H_16_	100.125	Alkane	2.94	98.38	46	4.4 × 10^−6^	0
Methyl Isobutyl Ketone	108‐10‐1	C_6_H_12_O	100.089	Ketone	2.65	116.5	19.9	6.9 × 10^−2^	17.1
2,2-Dimethoxybutane	3453‐99‐4	C_6_H14O2	118.099	Alkane	NA	99.8	43.3	NA	18.5
1-Nonene	124‐11‐8	C_9_H_18_	126.141	Alkene	3.75	146.9	5.40	1.1 × 10^−5^	0
5-Hepten-2-one, 6-methyl-	110‐93‐0	C_8_H_14_O	126.104	ketone	133	96	47	2.5 × 10^−1^	17.1
Nonane	111‐84‐2	C_9_H_20_	128.157	Alkane	12.1	150.47	4.45	1.6 × 10^−6^	0
D-Limonene	5989‐27‐5	C_10_H_16_	136.125	Terpene	8.76	177.6	1.98	7.3 × 10^−4^	0
Decane	124‐18‐5	C_10_H_22_	142.172	Alkane	NA	173	1.43	1.1 × 10^−6^	0
Undecane	1120‐21‐4	C_11_H_24_	156.188	Alkane	196	195	0.41	4.4 × 10^−4^	0
Isopropyl palmitate	142‐91‐6	C_19_H_38_O_2_	298.287	Ester	187	160	0.7	NA	26

The recovery of the VOCs depends in part on the emission rate of the compound from skin. Skin VOC emission rates are poorly investigated, nonetheless some values have been reported and included in table 1, which were between 0.01 and 1000 fmol × cm^−2^ × min^−1^. Operational sampling conditions also affect analyte recovery, such as sampling time, sampling temperature, and sorbent type, which were investigated below to determine an optimal sampling methodology to capture skin VOCs.

### 3.3. Optimization of sampling time based on PDMS sorbent

**Figure 2.**
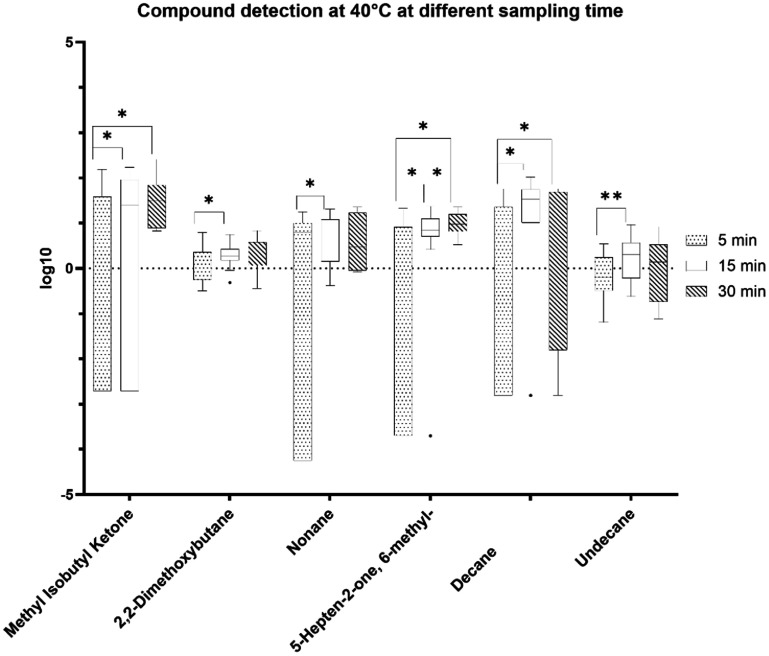
Comparison of 5-, 15-, and 30-minute sampling times on skin VOC recovery (40 °C sampling temperature with PDMS sorbent). Longer sampling times generally resulted in higher VOC recovery and decreased variability. 15 min was chosen as the optimal sampling time as it had sufficient VOC recovery with minimal burden to the volunteer. (*p = <0.05, **p = <0.005).

Three sampling times (5, 15, and 30 min) were investigated to identify the shortest sampling time that offered sufficient detection of selected compounds. Figure 2 shows the differences in the mean intensities of six select compounds. While sampling time was adjusted, sampling temperature was held constant at 40 °C, and PDMS sorbent was used.

Overall, with longer sampling times, analyte abundance typically increased, and variability decreased. For example, methyl isobutyl ketone, with a reported relatively low emission rate (2.65 fmol × cm^−2^ × min^−1^), was only detected in 8 out of 20 samples with 5 min of sampling, but was found in 14 samples when the sample time increased to 15 min. At 30 min this same analyte was measured in all samples and the variability decreased. In a similar way, nonane shows very low to no detection for multiple samples at 5 min of sampling duration. Interestingly, with 15 min, the amount detected increased by tenfold while variability decreased. The differences on intensities detected were statistically significant (*p* < 0.05) between 5 and 15 min.

For a compound with an intermediary emission rate value like 5-hepten-2-one 6-methyl, the detection at 5 min in is still lower and with a wider range of detection compared to the two other sampling times. In this case, statistical differences were found between all the studied times (5–15, 5–30 and 15–30 min). Undecane, with the highest emission rate of 196 fmol × cm^−2^ × min^−1^, has been detected though all the samples collected with low variability even at 5 min. Significant statistical differences were only found between 5 and 15 min.

Unfortunately, emission rate data are unavailable from previous studies for decane and 2,2-Dimethoxybutane. In the case of decane, its detection fluctuated, at 5 min of sampling the compound was detected only in 6 samples, at 15 min of sampling it was detected in all the samples, and at 30 min detected in only 17 of the 20-sample collected. 2,2-Dimethoxybutane was detected in all human volunteers for all sampling times expect at 5 min of sampling with 2 samples. This VOC has been detected in all sampling time with low variability even at the shortest sampling time, suggesting it could have a similar an emission rate such as undecane (±195 fmol × cm^−2^ × min^−1^).

Considering these results, it was determined that 5 min of sampling time from the skin was too short for an optimal detection of compounds, and, in general, sampling skin VOCs for 30 min rarely increased recovery when compared to just 15 min of sampling. 15 min were selected as the optimal sampling time for general skin VOC recovery.

### 3.4. Optimization of sampling temperature based on PDMS sorbent

Another way to classify VOCs can be based on their volatility properties, expressed by the vapor pressure (Vp) and boiling point (Bp) values (table 1), which ranged from 50 to 260 °C at atmospheric pressure. These two properties are inversely correlated, meaning that if the boiling point of a certain compound is low, then its vapor pressure is high and so is its volatility. It was suspected that some VOCs produced by the skin may be insufficiently volatile at room temperature. Therefore, an increase of HS temperature during skin sampling was tested to improve the detection of compounds with low vapor pressure.

**Figure 3.**
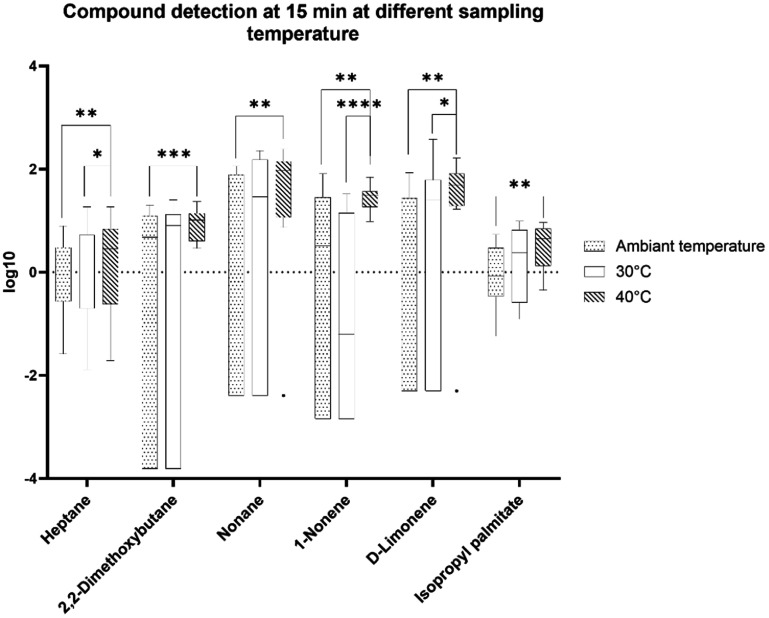
Comparison of three sampling temperatures on skin VOC recovery: ambient (no device heating), 30 °C, and 40 °C. In all cases, the sampling time was 15 min collected onto PDMS sorbent. Generally, there were no significant differences between ambient and 30 °C sampling; but 40 °C samples were selected as the optimal sampling temperature as it exhibited increased VOC abundances and decreased variability between volunteers. (*p = <0.05, **p = <0.005, ***p = <0.0005, ****p = <0.00005).

Three different sampling temperatures, ambient (no heating of the device), 30 °C, and 40 °C, were investigated (figure 3). Without heating, the temperature within the skin patch varied between 27 and 29 °C, and higher temperatures of 30 °C and 40 °C were reached though the combination of the two heated compounds within the device, the heated line and top heater sheet (figure 1(A)). For a sampling temperature of 30 °C the heating line and the top heater were set at 50 °C and 40 °C respectively, and for a sampling temperature at 40 °C the two heaters were set 60 °C and 50 °C. While temperature was adjusted, sampling time was held constant at 15 min, and PDMS sorbent was used.

No statistical differences were found between ambient temperature and 30 °C, which was expected since the temperature housing in both cases was similar (difference less than 3 °C). Also, for these lower temperatures, the variability between volunteers were higher, in part because these compounds were not always detected at lower temperatures.

When comparing 30 °C to 40 °C, statistical differences were found for heptane,1-nonene, and D-limonene, but not for 2,2-dimethoxybutane, nonane, and isopropyl palmitate, probably due to their high emission rate and high boiling point. This efficiency of the temperature effect during sampling is even more noticeable when comparing ambient temperature to 40 °C samples, resulting in significant statistical differences for all compounds. Interestingly, the highest statistical differences are found with the compounds with lowest boiling point (99 °C), 2,2-dimethoxybutane Noticeably, variability of a given compound decreased between volunteers as the sampling temperature increased. In this case, it was determined that skin sampling VOCs at 40 °C allows a better detection of a wider range of compounds with a reduction in variability. Consequently, 40 °C was selected as the optimal sampling temperature for general skin VOC sampling.

### 3.5. Comparison of Tenax TA and PDMS sorbent

After optimizing sampling time (15 mins) and temperature (40 °C), two different sorbent materials, PDMS and Tenax TA, were tested to demonstrate their efficiency to adsorb VOCs coming from the skin. PDMS is known for its highly hydrophobic properties whereas Tenax has affinity for compounds with higher polarity.

**Figure 4.**
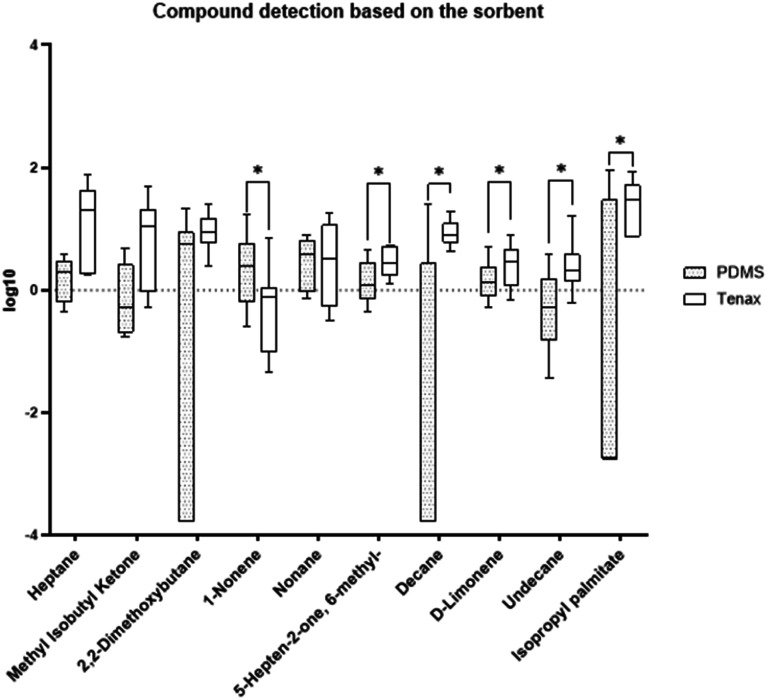
Comparison of VOC recovery by sampling onto PDMS and Tenax TA sorbents (sampling time 15 mins, sampling temperature 40 °C). Tenax TA demonstrated higher VOC abundances and decreased variability, so it was chosen as the optimal sorbent type for skin analysis. The normalized intensities were plotted, and statistical analysis performed (*: *p* = <0.05).

In this case (figure 4), statistical differences were mainly found for more polar compounds and higher molecular weight VOCs, such as nonanal, decanal, dodecyl acrylate and isopropyl palmitate. Also, compounds with high boiling points, such as decane and dodecane, presented statistical differences between materials, with Tenax showing higher intensities. From all the compounds detected with statistical differences between sorbent material, Tenax showed higher intensities than PDMS. Moreover, Tenax samples generally showed lower variability between samples than PDMS. Thus, Tenax TA was selected as the optimal sorbent based on these results.

### 3.6. Final sampling methodology and typical skin VOC profile

**Figure 5.**
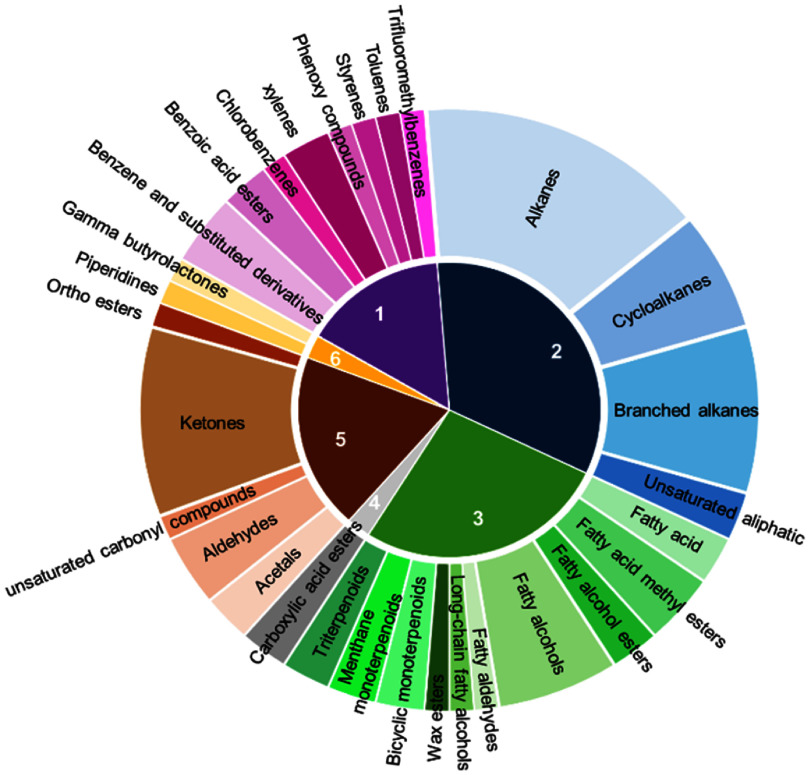
The chemical classes of skin VOCs as detected in 20 healthy volunteers. The inner circle represents the superclass (1: benzenoids, 2: hydrocarbons, 3: lipids and lipid-like molecules, 4: organic acids and derivates, 5: organic oxygen compounds, 6: organoheterocyclic compounds).

As described above, the final and optimized sampling conditions included 15 min of sampling at 40 °C using Tenax TA sorbent. With these optimal parameters additional sampling were done on 20 healthy volunteers. After data processing a list of 79 compounds included the 10 compounds prior selected for the optimization steps were extracted. Analysis of the detected VOCs following thermal desorption GC-MS revealed a variety of classes of compounds emanating from the skin (figure 5). The most representative chemical classes are alkanes, esters, ketones, alcohols, and aldehydes with respectively representing 32%, 16%, 10%, 10%, and 8% of the total number of compounds. Within the total 79 detected compounds some of the most frequently reported volatiles recovered from skin include nonanal, decanal, 5-hepten-2-6-methyl, hexanal, decane, and octane.

Many pollutants were also detected, such as BTEX, a mixture of benzene (found in *n* = 16 of the 20 samples), toluene (*n* = 20), ethylbenzene (*n* = 13), and xylenes (p-xylene *n* = 14, o-xylene *n* = 18). These carcinogens are commonly observed environmental exposure hazards, produced mostly by industrial activities and petroleum processes. These chemicals are categorized as hazardous pollutants and carcinogens by the United States Environmental Protection Agency (US EPA, 2017) and associate to numerous diseases by the World Health Organization (WHO). Thereby, our skin sampling device may be suited for environmental exposure assessment. Is it noteworthy that BTEX has been reported in more than 80% of the sunscreen sold within the US market [26] and as impurities in hand sanitizers and hygiene products; they were recovered in our sampling device despite the forearm being cleaned with ethyl alcohol prior to VOC sampling.

**Figure 6.**
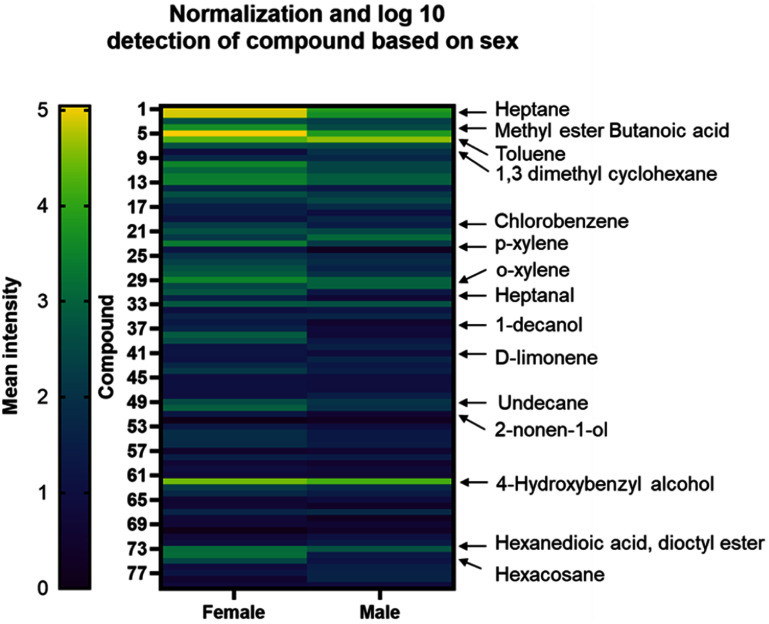
Heat map of mean compound detection of 79 skin VOCs between female (*n* = 7) and male (*n* = 13) volunteers. The scale 0–5 represent the detection intensity of compound with the blue lowest to yellow highest intensity.

Additionally, mean intensities by sex group were examined (figure 6). Highest intensity detected compounds in females were methyl isobutyl ketone, 2-pentanol, heptane, 4-hydroxybenzyl alcohol and toluene. In males the highest intensity detected were, were toluene, 4-hydroxybenzyl alcohol, 2-pentanol, methyl isobutyl ketone and heptane, in order. Three of the listed VOCs that showed significant discriminative differences between sex of the subjects were heptanal, methoxy-phenyl-oxime and 2,3,3-trimethylpentane, all found with higher intensities in females.

**Figure 7.**
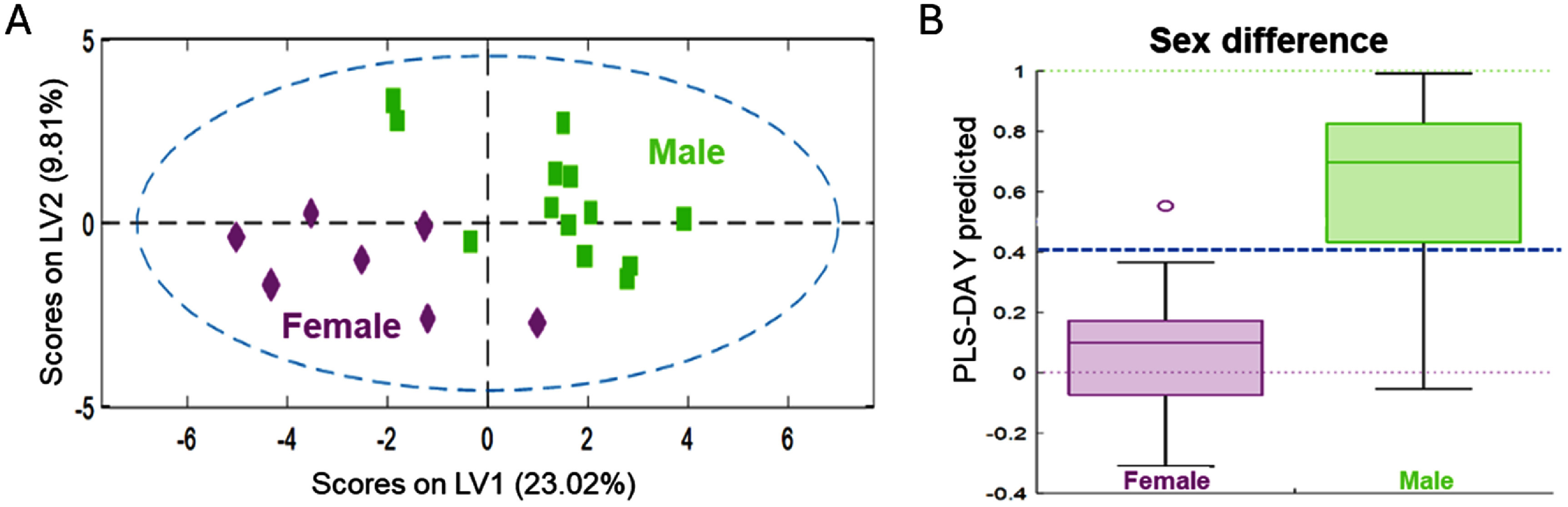
Representation of sex-based model by PLS-DA. (A). PLS-DA scores showing sample discrimination by sex between the first two Latent Variables (LV). (B). Y prediction values for female and male cohorts, with the model classification threshold identified as a blue dashed line.

As the purpose of this report is to showcase the feasibility of using our device in human studies, we did not control for many biological factors among our subjects. One available variable known was the biological sex of the volunteers, and even though we had a small cohort, we investigated whether we could observe any differences based on biology. Our PLS-DA model resulted in showed 17 VIP compounds, which explain, here the differences between sex groups, including heptane, butanoic acid methyl ester, methyl isobutyl ketone, cyclohexane, 1,3-dimethyl-octane 3-methyl-, phenylethyne, heptanal, 1-decanol, pentane, 2,3,3-trimethyl decane, cyclohexene, 4-methylene-1-(1-methylethyl)-, D-limonene; undecane; 2-methoxy-1,3-dioxolane; 2-nonen-1-ol, hexanedioic acid bis(2-ethylhexyl) ester, and hexacosane. Separation between groups by sex is represented in figure 7(A). The built model has a specificity of 0.75 and a sensitivity of 1.00 with an area under the curve equal to 0.80. PLS-DA predicted values for the two studied groups are represented in figure 7(B), showing a classification ability of the model to determine samples by sex. Samples from the male group were more misclassified than female samples, which could be explained by the higher detected intensities in females. While we do not make any claims that skin VOC emissions differs between females and males, we did observe these differences in our limited cohort.

These preliminary results provide a baseline of skin VOC emissions from 20 volunteers with no known health disorders, as measured by our new technology. As mentioned in the introduction, the origins of our reported VOCs may derive from a combination of sources, such as gland secretions or the skin microbiome. Future studies can use our device to explore skin VOC origins in real life studies or *in ex vivo* skin models. We hope to use this device to explore the possibility of using skin as a diagnostic biospecimen, as is being investigated in exhaled breath. Today, service dogs can be trained to alert handlers about blood sugar changes related to diabetes, or impending seizures for people with epilepsy. We suspect that body odor emissions may contribute to this phenomena, implying that wearable technologies could reduce the cost burden associated with medical alert service dogs.

Several limitations of our study should be acknowledged. First, our optimization study was limited to the parameters tested; use of other sorbents beyond PDMS or Tenax TA may yield different findings. Also, our reported skin VOC observations are limited to the smaller sample size (*n* = 20 volunteers), which is insufficient to properly characterize the skin volatilome. We also only sampled the posterior forearm area, which has different emissions than other parts of the body. Additionally, intravariability among volunteers cannot be overlooked, as skin emissions can vary over time or time of day, such as from fasting or disease state. These factors were not controlled in our experiments, as we focused on the feasibility of using our device in clinical studies. Finally, while our device’s gentle heating was found to release more VOCs from skin, some skin chemicals may still not be volatile enough, or may adsorb onto surfaces of the sampling device, and thus be missed.

## 4. Conclusion

In this study, we present a device to sample VOC emanations from human skin. While presented as posterior forearm sampler, the device design can be modified and scaled to sample other areas. We believe to be the first to present a skin sampling technology that includes a heating dimension, which we demonstrate to improve the recovery and decrease variability of skin VOC detection. Through an optimization study, we found that 15 min of sampling time at 40 °C onto Tenax TA sorbent yields optimal skin VOC recovery with minimal burden to the wearer. In a study of 20 healthy volunteers, we reported the observed abundances of 79 compounds represented mainly by alkanes, esters and ketones as the most representative chemical classes, and report our observed differences between female and male volunteers. BTEX pollutants were also detected from skin samples, providing an opportunity to use this technology for individual exposure assessment to environmental hazards. Future work seeks to use this technology to investigate skin VOC sampling.

## Data Availability

The data cannot be made publicly available upon publication because the cost of preparing, depositing and hosting the data would be prohibitive within the terms of this research project. The data that support the findings of this study are available upon reasonable request from the authors.
